# Concomitant malnutrition and frailty are significant risk factors for poor outcome following two-stage revision for chronic periprosthetic joint infection

**DOI:** 10.1186/s13018-023-04293-4

**Published:** 2023-10-27

**Authors:** Tengbin Shi, Zhi Chen, Dingxiang Hu, Dingwei Wu, Zhenyu Wang, Wenge Liu

**Affiliations:** 1 Department of Orthopedics, Fujian Medical University Union Hospital, Fuzhou, 086-350001 China; 2 Department of Rehabilitation Therapy, Jiangsu Rongjun Hospital, Wuxi, 214000 Jiangsu China

**Keywords:** Periprosthetic joint infection, Two-stage revision, Frailty, Malnutrition, Albumin, Outcomes

## Abstract

**Background:**

Two-stage revision remains the gold standard for periprosthetic joint infection (PJI) treatment. Although previous studies have examined malnutrition and frailty independently, their cumulative effects are not clear. Therefore, this study aimed to assess the individual and combined influence of malnutrition and frailty on the two-stage revision surgery.

**Methods:**

Patients with chronic PJI undergoing two-stage revision were retrospectively included. The definition of PJI is completely consistent with the evidence-based definition of PJI recorded by the MSIS in 2018. Preoperative serum albumin levels and 11-item modified frailty index scores were collected. Four cohorts were created: (1) Normal (N), (2) Frail (F), (3) Malnourished (M), and (4) Malnourished and frail (MF). Demographic data, comorbidities, and postoperative complications were collected and compared between the four cohorts.

**Results:**

A total of 117 consecutive patients were enrolled, 48% of patients were healthy (27.4% F, 16.2% M, and 9.4% MF). MF group showed lower scores on the physical composite scale of the 12-item short-form health survey (SF12-PCS), mental composite summary (SF12-MCS), Harris hip score (HHS), and knee society score (KSS) (*P* < 0.05). The incidence of reinfection in the MF group was higher than that in all other groups (MF vs. N; odds ratio [OR] 3.7, 95% confidence interval [CI] 1.37 − 8.82, *P* = 0.032). The incidence of complications in the MF group was higher than that in all other groups (MF vs. N; OR 4.81, 95% CI 1.58–9.26, *P* = 0.018). Postoperative transfusion events (OR 2.92, 95% CI 1.27–3.09, *P* = 0.021), readmission at 60 days after the operation (OR 4.91, 95% CI 1.82–13.80, *P* = 0.012) was higher in the MF patients. In addition, the extended length of stay after the operation was highest in the MF patients, with an OR of 5.78 (95% CI 2.16–12.04, *P* = 0.003).

**Conclusion:**

The concurrent presence of concomitant malnutrition and frailty in patients with PJI is related to poor prognosis and may be a predictor of the efficacy of two-stage revision. Future research will be needed to describe the benefits of improving these risk factors for patients with PJI.

## Introduction

Periprosthetic joint infection (PJI) is one of the most devastating and costly complications following primary total joint replacement (TJA), the reported incidence of PJI is approximately 2–3% [1–3], the combined annual hospital costs related to PJI of the hip and knee were estimated to be $1.85 billion by 2030 [4]. Two-stage revision remained the most widely performed treatment for PJI (the first stage is complete removal of all components, aggressive debridement, and placement of an antibiotic-loaded cement spacer, and the second stage is reimplantation of a new prosthesis after infection eradication) with a high rate of infection control [5–7]. Despite significant advances in surgical technique, these operations' reinfection and postoperative complications still occurred significantly [8, 9]. Therefore, to improve the prognosis of patients with PJI, it is crucial to define the preoperative risk assessment of complications following revision surgery for patient counseling and preoperative planning.

Recently, the effects of malnutrition on clinical outcomes following orthopedic surgeries have gradually attracted the attention of researchers. The literature reported a high incidence of malnutrition among primary TJA patients, ranging from 8.5 to 50% [10]. Furthermore, a growing body of evidence suggested a close relationship between malnutrition and various wound complications, such as delayed wound healing, persistent wound drainage, wound dehiscence, and surgical site infections [11]. Due to overconsumption of nutrition, pathological states such as chronic infections and tumors have been shown to induce a higher incidence of malnutrition [12, 13]. Given the preceding facts, the potential significance of malnutrition in PJI, a chronic consumptive infectious disease, may deserve even greater concern. Green et al. [14] identified low preoperative serum albumin as an independent risk factor for failure following first-stage resection in planned two-stage revision for PJI. A variety of methods used for diagnosing malnutrition have been proposed in the literature, including the use of serologic laboratory values (albumin < 3.5 g/dL, total lymphocyte count < 1500 cells/mm^3^, transferrin < 200 mg/dL, Zinc < 95 ug/dL), anthropometric measurements (calf muscle circumferences < 31 cm, arm muscle circumference < 22 mm, and the presence of triceps skin fold), and standardized nutrition score tools (Rainey–MacDonald nutritional index, the Mini Nutritional Assessment, and the Schwarzkopf nutritional index) [15].

Frailty is a complex syndrome characterized by an age-related decline in physiological capacity and an excessive vulnerability of the individual to endogenous and exogenous stressors, which can measure the physiological reserve and cumulative defects of several organ systems [16, 17]. Frailty may explain the observed heterogeneity of postoperative outcomes in some elderly patients, especially those who do not tolerate even mild stressors. Recent evidence shows that frailty is significantly associated with postoperative adverse events in patients receiving primary THA or TKA [18–20], and preoperative frailty is also identified as a risk factor for PJI mortality [21]. The modified frailty index (mFI) is a simplified form of the Canadian Study of Health and Aging (CSHA) Frailty Index, which has been shown to correlate independently with postoperative morbidity, mortality, and length of hospital stay (LOS) in different surgical populations, such as orthopedics, general surgery, and vascular surgery [20, 22, 23].

Malnutrition and frailty define a new high-risk cohort, whose physiological damage states have been reported to increase the incidence of complications and mortality after a variety of operations [24–26], including joint arthroplasty, however, it has not been reported in PJI. Patients with PJI usually have poor overall general condition and require prolonged antibiotic treatment and multiple surgeries [1], leading to increasing difficulty during two-stage reconstruction. Although previous studies have made great efforts to describe the risk factors for complications after two-stage revision in patients with PJI, concerns remain about the outcome, postoperative complications, and adverse events of this procedure. The additive effects of co-complications are still poorly understood and therefore, such a cohort must be recognized to prevent potentially catastrophic outcomes.

This study aimed to investigate the individual and combined effects of malnutrition and frailty on outcomes and complication rates in patients with chronic PJI after the second stage of two-stage revision. We hypothesized that the combination of malnutrition and frailty is associated with poorer prognosis and higher complication rates after the second stage of two-stage revision compared with these two entities alone.

## Materials and Methods

All consecutive patients from two tertiary care university hospitals scheduled to have two-stage revision for chronic PJI following primary knee or hip arthroplasty between January 2016 and December 2020 were enrolled. Our Institutional Review Board (IRB) approved this study, and patient informed consent was waived for this retrospective study. The definition of PJI is completely consistent with the evidence-based definition of PJI recorded by the MSIS in 2018 [27], which consists of 1 of the 2 major criteria (sinus tract communicating with joint or at least 2 positive periprosthetic culture results) or 4 of the 6 minor criteria (elevated C-reactive protein (CRP) level and erythrocyte sedimentation rate (ESR), elevated synovial fluid white blood cell (WBC) count or ++ change on a leukocyte esterase test strip, elevated synovial fluid polymorphonuclear (PMN), positive histological analysis of periprosthetic tissue, presence of purulence in the affected joint, a single positive culture). Chronic PJI was defined as the persisting PJI symptoms more than 4 weeks after the index arthroplasty [28]. The following patients were excluded: patients without known preoperative albumin levels; patients with missing outcome data or without regular follow-up data; patients with autoimmune disease, previous or concomitant therapy with corticosteroids or immunosuppressive drugs, malignant tumors, chronic liver diseases, renal diseases, and mental disorders; patients with non-PJI-related death.

The following data were collected from electronic patient files: age, sex, body mass index (BMI), comorbidities, alcohol intake, smoking status, concurrent medications, American Association of Anesthesiologists (ASA) scores, Charlson comorbidity index (CCI), laboratory tests, bacterial culture results, histology results, the index surgery type, and date, time from index surgery until infection, surgery-related data such as antibiotic prophylaxis, operation time, anesthesia, and blood loss in the first and second surgical stage.

Preoperative serum albumin quantification within 3 days before the two-stage revision was collected and based on previously reported threshold [29], patients with serum albumin levels < 3.5 g/dL were defined as hypoalbuminemia (malnourished) and > 3.5 g/dL as normoalbuminuric.

The present study used the mFI described by Saxton and Velanovich [30], which is an 11-variable assessment (Table 1) that maps 16 NSQIP variables to 11 variables in the CSHA Frailty Index. An mFI score was calculated by dividing the number of deficits present by the total number assessed (*n*/11). By prior literature, frail patients are defined as having mFI > 0.21 in compliance with precedence from prior literature [31]. Patients were divided into four cohorts based on their albumin levels and mFI scores, patients with normal albumin levels and mFI < 0.21 (normal, N), hypoalbuminemia and mFI < 0.21 (malnourished only, M), mFI > 0.21 and nor-hypoalbuminemia (frail only, F), and mFI > 0.21and hypoalbuminemia (combined frail and malnourished, MF).

**Table 1:** Variables of the modified frailty index (mFI)

1. History of diabetes mellitus
2. Functional status 2 (not independent)
3. History of chronic obstructive pulmonary disease or pneumonia
4. History of congestive heart failure
5. History of myocardial infarction
6. History of percutaneous coronary intervention, stenting, or angina
7. History of hypertension requiring medication
8. History of peripheral vascular disease or ischemic rest pain
9. History of impaired sensorium
10. History of transient ischemic attack or cerebrovascular accident
11. History of CVA with neurological deficit

### Surgical strategies

All operations were performed by the same surgical team in the two hospitals and were performed under general or spinal anesthesia, and the original incision was used. Synovial fluid and periprosthetic tissues were routinely collected for pathogenic microorganism culture and histology. The first stage of the two-stage revision included: the complete removal of all components, complete debridement, and implantation of an antibiotic-loaded spacer, then patients were given antibiotics treatment (2 weeks of intravenous antibiotic and 4–6 weeks of oral suppressive antibiotic) based on postoperative culture and drug sensitivity results decided by at least two orthopedic experts and one infectious disease expert. If the culture result was negative, vancomycin and meropenem were empirically employed. After infection control, the second stage includes the removal of the spacer, re-debridement, and implantation of a new prosthesis. Drainage tubes are generally not placed except for special needs. When antibiotic bone cement is needed, the formula is 2 g of vancomycin per 40 g of cement.

### Outcome and complication data

Following perioperative events in the second surgical stage were recorded: reinfection, wound dehiscence, unplanned second surgery for any reasons, unplanned blood transfusion, wound dehiscence, myocardial infarction, cardiac arrest, unplanned intubation, stroke, shock, pulmonary embolism, deep vein thrombosis, pneumonia, sepsis, urinary tract infection, acute kidney injury. Extended length of stay (defined as the postoperative length of stay beyond the 75th percentile), 30-, 60-, and 90-day readmission rates, and 1-year mortality were also recorded.

Successful eradication was defined as an improvement of clinical symptoms, a healed wound without sinus tracts, drainage, or pain, no infection recurrence caused by the same organism strain, no further need for surgical intervention, and no occurrence of PJI-related mortality. The diagnosis of infection recurrence was based on the symptoms, signs, laboratory tests, and images.

Patients were followed up in an outpatient clinic at least at 1 month, 3 months, 6 months, and 12 months after the surgery during the first year and every 6 months afterward. And patient-reported outcomes (PROMs) including the Visual Analog Scale (VAS), the physical composite Scale (PCS), mental composite summary (MCS) scores from the 12-item short-form health survey (SF12), Harris hip score (HHS), and knee society score (KSS) were collected at every follow-up visit for pain and joint function assessment.

### Statistical analysis

Statistical analysis was performed in SPSS, version 25 (IBM Corporation, Armonk, NY, USA). Continuous variables (including BMI, operative time, intraoperative blood loss, follow-up time, HHS scores, KSS scores, SF-12 scores, and VAS scores) were presented as mean (standard deviation (SD)). Categorical variables (including joint involved, gender, smoker, alcohol consumption, ASA grade, history of surgery for PJI, antibiotic prophylaxis, presence of sinus, known organism, and anesthesia type) were presented as frequency. Differences in patient and clinical characteristics and clinical outcomes (SF-12, HHS, KSS, and VAS) were assessed using the chi-squared test or Fisher's exact test for categorical variables and the one-way ANOVA or Mann–Whitney U test for continuous variables. Univariate analysis was conducted to assess the complication and adverse events rate between the following groups: (1) Normal (N), (2) Frail (F), (3) Malnourished (M), and (4) Malnourished and frail (MF), and then a multivariate binary logistic regression model (with reinfection, re-revision for aseptic reasons, transfusion, any complications, all-cause unscheduled readmissions at 30, 60, 90 days, 1-year mortality, and extended LOS as binary outcomes) was used to adjust for the following factors (with statistical significance in Table 2): age, BMI, ASA grade at the first stage, operative time and intraoperative blood loss of the first stage, operative time and intraoperative blood loss of the second stage, to generate an odds ratio (OR) with a 95% confidence interval (CI). Our model compared complications, mortality, and adverse events using normal (N) patients as the normative reference. MF, F, and M cohorts were used as the experimental groups. Secondary analysis examined the MF cohort compared to the F-only and M-only groups to quantify the risks of combined malnourished and frailty, relative to either issue alone. *P* values less than 0.05 were considered statistically significant.

**Table 2:** Demographic details and surgical data

Parameters	Normal	Frail^a^	Malnourished^b^	Frail and malnourished^c^	*P* value
Number of patients	55 (47.0%)	32 (27.4%)	19 (16.2%)	11 (9.4%)	–
Joint involved, *n* (%)					0.941^†^
Hip	35 (63.6%)	19 (59.4%)	13 (68.4%)	7 (63.6%)	
Knee	20 (36.4%)	13 (40.6%)	6 (31.6%)	4 (36.4%)	
Laterality (left), *n* (%)	29 (52.7%)	17 (53.1%)	9 (47.4%)	5 (45.5%)	0.953^†^
Age, years					**0.035**^**†**^
≥ 65	41 (74.5%)	19 (59.4%)	9 (47.4%)	4 (36.4%)	
< 65	14 (25.5%)	13 (40.6%)	10 (52.6%)	7 (63.6%)	
Gender (female), *n* (%)	29 (52.7%)	18 (56.3%)	12 (63.2%)	6 (54.5%)	0.903^†^
BMI, kg/m^2^ (SD)	30.3 (6.4)	29.8 (5.9)	30.6 (6.8)	31.8 (6.2)	< 0.001*
Current smoker (yes), *n* (%)	7 (12.7%)	5 (15.6%)	3 (15.8%)	2 (18.2%)	0.899^†^
Alcohol consumption, *n* (%)	12 (21.8%)	5 (15.6%)	4 (21.1%)	1 (9.1%)	0.824^†^
ASA grade at the first stage, *n* (%)					**0.026**^**†**^
I	8 (14.5%)	2 (6.3%)	6 (31.6%)	0	
II	29 (52.7%)	17 (53.1%)	8 (42.1%)	5 (45.5%)	
III	18 (32.7%)	11 (34.4%)	4 (21.1%)	3 (27.3%)	
IV	0	2 (6.3%)	1 (5.3%)	3 (27.3%)	
History of surgery for PJI	12 (21.8%)	8 (25.0%)	4 (21.1%)	4 (36.4%)	0.755^†^
First stage					
Antibiotic prophylaxis	29 (52.7%)	14 (43.8%)	11 (57.9%)	7 (63.6%)	0.637^†^
Presence of sinus, *n* (%)	13 (23.6%)	9 (28.1%)	6 (31.6)	3 (27.3%)	0.905^†^
Known organism, *n* (%)	37 (67.3%)	17 (53.1%)	12 (37.5%)	7 (63.6%)	0.635^†^
Operative time, min (SD)	152.8 (42.3)	158.6 (48.5)	156.9 (46.5)	161.2 (48.8)	< 0.001*
Intraoperative blood loss (mL)	776.7 (632.0)	786.7 (663.3)	782.1 (665.9)	789.3 (686.6)	< 0.001*
Anesthesia type, *n* (%)					0.720^†^
General	32 (58.2%)	19 (59.4%)	13 (68.4%)	7 (63.6%)	
Spinal	20 (36.4%)	11 (34.4%)	5 (26.3%)	2 (18.2%)	
Other	3 (5.5%)	2 (6.3%)	1 (5.3%)	2 (18.2%)	
Second stage					
The second stage completed	43 (78.2)	21 (65.6%)	12 (63.2%)	8 (72.7%)	0.450^†^
Positive culture, *n* (%)	8 (18.6%)	6 (28.6%)	3 (25.0%)	2 (25.0%)	0.944^†^
ASA grade at the second stage, *n* (%)					0.423^†^
I	6 (14.5%)	2 (6.3%)	3 (31.6%)	0	
II	26 (56.4%)	13 (53.1%)	6 (42.1%)	4 (45.5%)	
III	11 (29.1%)	5 (34.4%)	2 (21.1%)	3 (27.3%)	
IV	0	1 (6.3%)	1 (5.3%)	1 (27.3%)	
Antibiotic prophylaxis	35 (81.4%)	16 (76.2%)	9 (75.0%)	7 (87.5%)	0.863^†^
Operative time, min (SD)	162.5 (45.2)	172.8 (45.7)	168.3 (49.3)	170.5 (42.6)	< 0.001*
Intraoperative blood loss (mL)	1086.7 (948.2)	1132.7 (1023.7)	1138.1 (1061.9)	1169.3 (1082.6)	< 0.001*
Mean follow-up, years (range)	38.3 (20.9)	38.8 (22.9)	37.8 (23.9)	39.3 (22.5)	0.813*

Bold values indicate that the corresponding *P*-value is less than 0.05

*ASA* American Society of Anesthesiologists, *BMI* body mass index, *IQR* interquartile range, *SD* standard deviation

*Independent-samples t test

^†^*Pearson chi-squared test or Fisher's exact test

^a^Frail: albumin > 3.5, mFI > 0.21

^b^Malnourished: albumin < 3.5, mFI < 0.21

^c^Frail and malnourished: albumin < 3.5 and mFI > 0.21

## Results

A total of 117 chronic PJIs (52 males and 65 females) met the inclusion criteria with an average age of 63.93 ± 8.62 (36–82) years, and an average BMI was 30.4 ± 6.3. These 117 individuals underwent an intended two-stage revision, and 84 (56 hips and 28 knees) completed the second stage. Forty-seven percent of patients were not frail or malnourished (47.0%), 27.4% and 16.2% of patients were frail-only and malnourished-only, respectively, and 9.4% of patients were classified as both frail and malnourished. Statistically significant differences between cohorts were detected regarding age, BMI, ASA grade at the first stage, operative time and intraoperative blood loss of the first surgery, and operative time and intraoperative blood loss of the second surgery (*P* < 0.05, Table 2).

### Patient-reported outcomes

At the last follow-up, 81(69.2%) patients completed the questionnaire, statistical analysis showed significant differences among the four cohorts in the PROMs including SF12-PCS, SF12-MCS, HHS, and KSS (*P* < 0.05, Table 3), and the VAS score did not show any significant difference (*P* = 0.231). When compared with the N group alone (Table 4), the M or F group didn’t show any significant difference except SF12-MCS (*P* = 0.030; *P* = 0.026), while the MF group showed a significant difference in SF12-PCS, SF12-MCS, HHS and KSS (*P* < 0.05).

**Table 3:** Comparison of patient-reported outcomes between four groups

	Normal	Frail	Malnourished	Malnourished and Frail	*P* value*
Total nonmissing, *n* (%)	38	22	13	8	–
SF12—MCS	38.30 (11.35)	34.85 (12.59)	35.27 (13.45)	32.43 (12.32)	**0.031**
SF12—PCS	55.82 (14.58)	52.08 (13.92)	53.64 (13.52)	50.65 (13.75)	**< 0.001**
VAS	2.12 (1.34)	2.23 (1.06)	2.09 (1.28)	2.31 (0.93)	0.231
HHS	77.84 (10.42)	75.47 (9.88)	75.21 (12.63)	72.45 (10.70)	**0.013**
KSS	74.14 (10.27)	71.26 (11.54)	72.41 (11.95)	68.95 (13.23)	**0.039**

Bold values indicate that the corresponding *P*-value is less than 0.05

*MCS* mental composite scale, *PCS* physical composite scale, *HHS* Harris hip score, *KSS* knee society score, *VAS* visual analog scale pain score, *SF12* 12-item short form health survey

*One-Way ANOVA

**Table 4:** Patient-reported outcomes of malnourished and frail, malnourished-only, or frail-only patients compared to normal patients

	Malnourished and frail	*P* value*	Malnourished-only	*P* value*	Frail-only	*P* value*
SF12—MCS	32.43 (12.32) versus 38.30 (11.35)	**< 0.001**	35.27 (13.45) versus 38.30 (11.35)	**0.030**	34.85 (12.59) versus 38.30 (11.35)	**0.026**
SF12—PCS	50.65 (13.75) versus 55.82 (14.58)	**0.016**	53.64 (13.52) versus 55.82 (14.58)	0.325	52.08 (13.92) versus 55.82 (14.58)	0.093
VAS	2.31 (0.93) versus2.12 (1.34)	0.885	2.09 (1.28) versus 2.12 (1.34)	0.983	2.23 (1.06) versus 2.12 (1.34)	0.961
HHS	72.45 (10.70) versus 77.84 (10.42)	**0.029**	75.21 (12.63) versus 77.84 (10.42)	0.692	75.47 (9.88) versus 77.84 (10.42)	0.716
KSS	68.95 (13.23) versus 74.14 (10.27)	**< 0.001**	72.41 (11.95) versus 74.14 (10.27)	0.762	71.26 (11.54) versus 74.14 (10.27)	0.652

Bold values indicate that the corresponding *P*-value is less than 0.05

*One-way ANOVA, pairwise comparisons using LSD test

### Perioperative complications and adverse events

Table 5 shows a univariate analysis of perioperative complications and adverse events in the four cohorts. The infection recurrence rate of MF patients is 45.5%, which is higher than that of the N patients (14.5%), F patients (10.9%), and M patients (31.6%) (*P* = 0.043). Any complications rate after the operation was higher in the MF group (63.6%), compared with the N group (20%), F group (21.9%), and M group (31.6%) (*P* = 0.029). The incidence of transfusion events, readmission at 30 days and 60 days after the operation, and extended length of stay also showed statistical differences. It is worth noting that the mortality within 1 year after operation was only 1.8% in the N group, rising to 6.25% in F patients, followed by 5.26% in M patients and 9.1% in MF patients, but with no statistical difference.

**Table 5:** Univariate analysis of perioperative complications and adverse events of the second surgery of two-stage revision in four groups

	Normal	Frail	Malnourished	Malnourished and frail	*P* value*
Reinfection, *n* (%)	7 (12.8%)	5 (10.9%)	6 (31.6%)	5 (45.5%)	**0.043**^†^
Deep infection	3 (5.5%)	1 (3.1%)	2 (10.5%)	2 (18.2%)	0.261^†^
Superficial infection	4 (7.3%)	3 (9.3%)	4 (21.1%)	3 (27.2%)	0.203^†^
Re-revision for aseptic reasons, *n* (%)	4 (7.3%)	5 (10.9%)	3 (15.8%)	2 (18.2%)	0.471^†^
Transfusion	6 (10.9%)	4 (12.5%)	3 (15.8%)	5 (45.5%)	**0.017**^**†**^
Any complications^a^	11 (20%)	7 (21.9%)	6 (31.6%)	7 (63.6%)	**0.029**^**†**^
30-day readmission, *n* (%)	3 (5.5%)	5 (15.6%)	3 (15.8%)	4 (36.4%)	**0.030**^**†**^
60-day readmission, *n* (%)	4 (7.3%)	6 (18.8%)	4 (21.1%)	4 (36.4%)	**0.047**^**†**^
90-day readmission, *n* (%)	3 (5.5%)	2 (6.3%)	1 (5.3%)	2 (18.2%)	0.436^†^
1-year mortality, *n* (%)	1 (1.8%)	2 (6.3%)	1 (5.3%)	1 (9.1%)	0.385^†^
Extended length of stay, *n* (%)	9 (16.4%)	12 (37.5%)	8 (42.1%)	7 (63.6%)	0.004*

Bold values indicate that the corresponding *P*-value is less than 0.05

*One-Way ANOVA

^†^Pearson chi-squared test or Fisher's exact test

^a^Complications including wound dehiscence, pulmonary embolism, deep vein thrombosis, pneumonia, myocardial infarction, cardiac arrest, sepsis, urinary tract infection, acute kidney injury

Multivariate analyses yielded similar results. In the multivariate model (Table 6), which controlled for confounders (patient demographic, surgical parameters), the odds of reinfection after the two-stage revision were highest in the MF patients, with an OR of 3.71 (95% CI 1.37–8.82, *P* = 0.032), while F or M patients showed no statistic difference. The odds of any complications after the operation were also higher in the MF patients, with an OR of 4.81 (95% CI 1.58–9.26, *P* = 0.018). The odds of the extended length of stay after the operation were highest in the MF patients, with an OR of 5.78 (95% CI 2.16–12.04, *P* = 0.003), followed by M patients (OR 3.72, 95% CI 1.34–11.83) and F patients (OR 3.07, 95% CI 1.22–4.43). Similarly, the odds of transfusion events (OR 2.92, 95% CI 1.27–3.09, *P* = 0.021), readmission at 60 days after the operation (OR 4.91, 95% CI 1.82–13.80, *P* = 0.012) was higher in the MF patients. Table 6 shows the complete OR data of the comparison between F, M, and MF patients to N patients.

**Table 6:** Multivariable analysis of postoperative complications and adverse events of the second surgery of two-stage revision in malnourished and frail, malnourished-only, or frail-only patients compared to normal patients

	Frail and malnourished		Malnourished		Frail	
	OR (95% CI)	*P* value	OR (95% CI)	*P* value	OR (95% CI)	*P* value
Reinfection	3.71 (1.37–8.82)	**0.032**	3.17 (0.91–11.06)	0.131	1.27 (0.37–4.40)	0.956
Re-revision for aseptic reasons	1.19 (0.17–8.45)	1.000	2.39 (0.48–11.83)	0.523	2.36 (0.59–9.53)	0.385
Transfusion	2.92 (1.27–3.09)	**0.021**	1.53 (0.34–6.84)	0.878	1.17 (0.30–4.49)	1.000
Any complications	4.81 (1.58–9.26)	**0.018**	1.85 (0.57–5.96)	0.349	1.12 (0.39–3.26)	0.835
30-day readmission	3.85 (0.56–16.38)	0.191	1.25 (0.10–9.86)	0.854	1.16 (0.18–7.31)	1.000
60-day readmission	4.91 (1.82–13.80)	**0.012**	3.25 (0.60–17.71)	0.172	3.21 (0.71–14.46)	0.231
90-day readmission	3.85 (0.56–16.38)	0.191	1.25 (0.10–9.86)	0.854	1.16 (0.18–7.31)	1.000
1-year mortality	0.31 (0.31–21.61)	0.308	3.00 (0.178–23.47)	0.450	3.60 (0.31–21.37)	0.552
Extended length of stay	5.78 (2.16–12.04)	**0.003**	3.72 (1.34–11.83)	**0.047**	3.07 (1.22–4.43)	**0.026**

Multivariable analysis was performed controlling for patient demographics, operative time, and intraoperative blood loss as presented in Table 2. Bold values indicate that the corresponding *P*-value is less than 0.05

*OR* odds ratio, *CI* confidence interval

There was no significant increase in the likelihood of reinfection in MF patients when compared with F or M patients (Table 7). When comparing odds of postoperative transfusion events, MF patients had 23% greater odds of postoperative transfusion events versus F patients (95% CI 1.20–7.41, *P* 0.031) and 44% increased odds versus M patients (95% CI 1.82–9.61, *P* = 0.018). MF patients also had a significantly greater odd of the extended length of stay compared with F patients (OR 1.66, 95% CI 1.20–7.41, *P* = 0.022) and M patients (OR 2.17, 95% CI 1.81–3.59, *P* = 0.026). Although there was no significant increase in the likelihood of any complication in MF patients when compared with M patients (*P* = 0.185), MF patients had 1.65 increased odds of any complication relative to F patients (95% CI 1.41–10.65, *P* = 0.029).

**Table 7:** Multivariable analysis of postoperative complications and adverse events of the second surgery of two-stage revision in malnourished and frail compared to frail-only or malnourished-only patients

	Malnourished and frail versus frail-only		Malnourished and frail versus malnourished-only	
	OR (95% CI)	*P* value	OR (95% CI)	*P* value
Reinfection	3.50 (0.98–20.63)	0.108	4.90 (1.20–11.93)	0.714
Re-revision for aseptic reasons	1.54 (0.20–6.30)	0.956	2.83 (0.45–9.83)	0.566
Transfusion	1.23 (1.20–7.41)	**0.031**	1.44 (1.82–9.61)	**0.018**
Any complications	1.65 (1.41–10.65)	**0.029**	1.81 (1.58–12.26)	0.185
30-day readmission	3.09 (0.65–14.62)	0.303	3.05 (0.54–12.37)	0.372
60-day readmission	2.48 (0.54–11.27)	0.436	2.14 (0.41–11.17)	0.417
90-day readmission	3.33 (0.41–18.13)	0.267	4.00 (0.32–50.23)	0.537
1-year mortality	1.50 (0.12–11.36)	0.133	5.42 (3.68–8.50)	0.815
Extended length of stay	1.66 (1.26–3.63)	**0.022**	2.17 (1.81–3.59)	**0.026**

Multivariable analysis was performed controlling for patient demographics, operative time, and intraoperative blood loss as presented in Table 2. Bold values indicate that the corresponding *P*-value is less than 0.05

*OR* odds ratio, *CI* confidence interval

## Discussion

Due to the increasing aging population and a sharp increase in TJAs, the number of PJIs is expected to grow exponentially over the next decade [3]. Two-stage revision though considered the gold standard is a technically demanding procedure, with a concerning failure rate [6]. The treatment failure not only causes physical and mental suffering to patients but also imposes a substantial burden on families and society [32]. To improve the prognosis of patients with PJI, there is a continued need to identify and correct modifiable risk factors preoperatively. Both malnutrition and frailty have been independently associated with adverse events following revision for PJI, however, there is little known about how malnutrition and frailty in this population interact. Our study observed a high incidence of malnutrition and frailty in patients receiving two-stage revision, leading to poorer postoperative outcomes and quality of life. Furthermore, patients with coexisting malnutrition and frailty had significantly higher incidence of postoperative complications and longer hospital stays than those with only one of them.

Recently, the effects of malnutrition on clinical outcomes following orthopedic surgeries have gradually attracted the attention of researchers. The literature reported that the incidence of malnutrition among primary TJA patients was about 8.5% [10]. More and more evidence suggests a close relationship between malnutrition and various complications, such as wound, cardiovascular, pulmonary, neurological, and renal complications [11, 33, 34]. Due to overconsumption of nutrition, a pathological state like chronic infections has been shown to induce a higher incidence of malnutrition [13]. The present study demonstrated that 16.2% of patients were malnourished, as defined by serum albumin of < 3.5 g/dL, higher than primary TJA and hip fracture [24, 26]. Several studies reported that the incidence of malnutrition in PJI patients was 2–3 times higher than in those with aseptic loosening [35, 36]. Some scholars have revealed that malnutrition reached up to 48.1–98.9% of patients undergoing the first stage of revision surgery [35, 37]. Given the preceding facts, the potential significance of malnutrition in PJI deserves even greater concern.

Frailty is considered a decline of reserve and function across several physiological systems, which has been established to be strongly associated with poor clinical outcomes [38, 39]. Previous studies demonstrated an average mFI of 0.09 in patients undergoing a primary TJA, and the risk of complications and mortality increased alongside increments of the mFI [19, 20]. Meyer et al. identified hospital frailty risk score (HFRS) as an independent risk factor for adverse outcomes in patients receiving primary THA [18]. Several studies revealed increased complication and readmission rates, worse function recovery, and prolonged hospitalization in patients with frailty than those with undetected frailty [40, 41]. Although frailty is considered a predictor of adverse outcomes for multiple surgeries, it has not been well studied in revision TJAs. Our study found that 27.4% of patients were frail, and frailty patients had significantly worse function outcomes than healthy counterparts.

It is worth noting that 9.4% of patients were both malnourished and frail, which was significantly associated with poorer function outcomes, higher complication rates, and longer hospital stays than those with only malnutrition or frailty. This finding suggested that the combination of malnutrition and frailty might be a better indicator for risk classification and outcome estimation than the usage of either malnutrition or frailty alone. Although many risk factors are difficult to optimize and preoperative intervention may be ineffective, preoperative correction of malnutrition is feasible and cost-effective [42]. Bohl et al. reported a significantly improved nutrition status at the second stage revision TJA (4.2–18.6% malnutrition) compared with the first stage (48.1–98.9% malnutrition) [35], suggesting malnutrition might be reversible. Torchia et al. identified that screening and treatment for malnutrition in TKA patients reduced the absolute risk of PJI by 0.07% [42]. In another study, Schroer et al. found that utilization of nutrition interventions in malnutrition patients undergoing TJA could significantly decrease the readmission rate, reducing the length of stay and hospitalization costs [43]. Similarly, it should be noticed that many aspects of frailty are also modifiable to some extent. Although not completely reversible, certain chronic diseases such as hypertension, diabetes, congestive heart failure, and chronic obstructive pulmonary disease can be medically controlled and optimized during the perioperative period through multidisciplinary collaboration, to keep them under optimal control to minimize their possible harmful effects on surgery outcomes. Under the proper circumstances, appropriate postponement of surgery in patients with a history of coronary artery disease, to lower the incidence of cardiac complications [44]. Careful preoperative assessment should be performed for asymptomatic peripheral vascular disease, and the use of tourniquets should be carefully considered during surgery to prevent arterial complications [45]. In addition, physical activity/exercise is considered one of the main strategies to counteract frailty-related physical impairment in the elderly [46], however, the implementation of physical activity/exercise in PJI patients is also contradictory and difficult, due to the loss of some joint functions. Even then, the paucity of proven frailty-targeted interventions after the identification of frailty is still a challenge. There is still a lack of sufficient evidence on the effectiveness of malnutrition and frailty interventions in PJI patients undergoing two-stage revision. Our study highlights the need for further research to investigate its efficacy and improve clinical outcomes for this vulnerable population.

Compared to other measures tools of nutritional status, the clinical utility of serum albumin levels is high for it is often used as one of the laboratory indicators routinely collected preoperatively, and can easily be applied with no additional cost to the patient or doctors. The mFI was the most reported tool for frailty assessment and it's been valid in large cohorts including the field of orthopedics [23], with precise and duplicable risk estimates. Besides, mFI can be easily applied without the need for special equipment or extensive chart review, special tests, and training, making it of great practical applicability in the clinical setting. However, mFI is currently limited to the 11 variables included. It does not include other variables that may affect frailty, such as sarcopenia, indeed, Velanovich et al. [22] first formulated the 11-Item mFI from the NSQIP database, they propose its universal use in all surgical patients irrespective of specialty. Thus, a consensus is urgently needed to standardize the quantitative method for frailty in patients with PJI.

Several limitations to this investigation must be recognized. First, the study design is retrospective, which is inherently limited to establishing a causal relationship between malnutrition, frailty, and adverse outcomes. Second, the sample size in this study was relatively small, which precluded some subgroup analyses but represents a large series available in PJI. Third, malnutrition was defined by low serum albumin alone in this study, although generally accepted, we did not evaluate other possible markers of malnutrition, such as prealbumin, total lymphocyte count, or transferrin, which could have increased the sensitivity of the true detection of malnourished patients. Future studies need more sophisticated and standardized screening protocols for malnutrition assessment. Fourthly, due to personal preference or current satisfied function with the temporary joint prosthesis spacer, only 71.8% (84/117) of the patients completed the two-stage revision, which may have influenced our conclusions. Lastly, our study only included data on hospitalized patients, so our results may underestimate some complications including mortality and reinfection. Future prospective studies with much larger sample sizes and longer follow-up periods are needed to confirm our current study findings.

## Conclusions

In conclusion, the combination of malnutrition and frailty was first reported to be associated with outcomes following the two-stage revision for chronic PJI. The concomitant malnutrition and frailty represent a previously undefined high-risk cohort in patients with chronic PJI, and it must be recognized that the potential for poorer outcomes in these patients is high, improving preoperative malnutrition and frailty may be the way forward in providing appropriate intervention for at-risk patients. The study can be utilized by clinicians to optimize both preoperative assessment and perioperative management to share preoperative decision-making with patients and their families and counsel patients about the potential complications following this procedure. Future studies focus on determining the impact of preoperative optimization of these high-risk cohorts on postoperative outcomes.

## Data Availability

The datasets used and/or analyzed during the current study are available from the corresponding author upon reasonable request.

## Abbreviations

PJI: Periprosthetic joint infection
MF: Malnourished and frail
TJA: Total joint replacement
mFI: Modified frailty index
CSHA: Canadian Study of Health and Aging
LOS: Length of hospital stay
BMI: Body mass index
ASA: American Association of Anesthesiologists
CCI: Charlson comorbidity index
PROMs: Patient-reported outcomes
VAS: Visual analog scale
PCS: Physical composite scale
MCS: Mental composite summary
SF12: 12-Item short-form health survey
HHS: Harris hip score
KSS: Knee society score
